# Single-cell network biology for resolving cellular heterogeneity in human diseases

**DOI:** 10.1038/s12276-020-00528-0

**Published:** 2020-11-26

**Authors:** Junha Cha, Insuk Lee

**Affiliations:** 1 Department of Biotechnology, College of Life Science & Biotechnology, Yonsei University, Seoul, 03722 Korea; 2 Department of Biomedical Systems Informatics, Yonsei University College of Medicine, Seoul, 03722 Korea

**Keywords:** Gene regulatory networks, Bioinformatics

## Abstract

Understanding cellular heterogeneity is the holy grail of biology and medicine. Cells harboring identical genomes show a wide variety of behaviors in multicellular organisms. Genetic circuits underlying cell-type identities will facilitate the understanding of the regulatory programs for differentiation and maintenance of distinct cellular states. Such a cell-type-specific gene network can be inferred from coregulatory patterns across individual cells. Conventional methods of transcriptome profiling using tissue samples provide only average signals of diverse cell types. Therefore, reconstructing gene regulatory networks for a particular cell type is not feasible with tissue-based transcriptome data. Recently, single-cell omics technology has emerged and enabled the capture of the transcriptomic landscape of every individual cell. Although single-cell gene expression studies have already opened up new avenues, network biology using single-cell transcriptome data will further accelerate our understanding of cellular heterogeneity. In this review, we provide an overview of single-cell network biology and summarize recent progress in method development for network inference from single-cell RNA sequencing (scRNA-seq) data. Then, we describe how cell-type-specific gene networks can be utilized to study regulatory programs specific to disease-associated cell types and cellular states. Moreover, with scRNA data, modeling personal or patient-specific gene networks is feasible. Therefore, we also introduce potential applications of single-cell network biology for precision medicine. We envision a rapid paradigm shift toward single-cell network analysis for systems biology in the near future.

## Systems biology: A single-cell, network-driven approach to disease

Gene regulatory networks reconstructed from single-cell RNA sequencing datasets are allowing researchers to better understand the molecular circuits and cell states that contribute to complex human disease. Junha Cha and Insuk Lee from Yonsei University in Seoul, South Korea, review the concept of ‘single-cell network biology’, which involves using computational algorithms on genetic expression data from thousands of cells to infer functional interactions in various biological contexts. This systems biology approach to analyzing the profiles of messenger RNA in single cells is helping researchers discover new signaling pathways that could serve as disease biomarkers or therapeutic targets. In the future, patient-specific models of personal gene networks could explain why certain genetic variants affect disease risk. This research could also eventually lead to new types of individualized medical treatments.

## Introduction

The adult human body is composed of ~37 trillion cells^1^, which are the functional units of organismal systems. Although each cell contains almost identical genomic information, at least several hundred major cell types with distinct morphology, behavior, and functions are expected to exist in the human body. Deviation from the destined identity of functional cells is a major cause of human diseases. Different cellular compositions of tumor tissue may result in different drug responses and prognoses. Disease-associated genetic variants affect only particular cell types, which makes functional validation of candidate variants derived from genome-wide association studies challenging^2^. Therefore, understanding human body operation at the cellular resolution is the ultimate goal in biology and medicine.

Investigation of individual cell types in vivo is technically challenging. Flow cytometry analysis has been used for single-cell profiling for the past several decades^3^, albeit with some limitations. First, it is a targeted analysis method for only a preselected set of molecules. Second, due to the spectral limitation of fluorescent proteins, this method can profile up to 17 proteins simultaneously, which is extended to ~40 proteins by mass cytometry^4^. Recently, we have witnessed a rapid improvement in single-cell RNA sequencing (scRNA-seq) technology, which is indeed a game changer in the field of single-cell biology. Current scRNA-seq technology can easily generate whole-transcriptome data for hundreds to thousands of cells from a single sequencing reaction and identify key genes associated with each cell type or state by differential expression analysis across distinct cellular groups of similar transcriptome. Therefore, we now characterize individual cell types or states in a tissue that is generally composed of diverse cell types. To date, a wide variety of methods for scRNA-seq data generation and analysis have been developed, and they are extensively described in other excellent reviews^4–7^. Recent benchmarking studies also showed that scRNA-seq protocols differ substantially in their ability to capture RNA, scalability, and cost effectiveness^8,9^.

Despite much improvement, single-cell omics may not be sufficient for understanding cellular heterogeneity. Although differential expression analysis of scRNA-seq data may identify genes specific to cell types and states, understanding cellular identity simply from a list of up or downregulated genes would be a daunting task because the functional effects of genes depend on their relationships. Gene functions and the effects of disease-associated variants are largely attributable to the interaction partners of these genes in the given cellular context^10,11^. From a systems biology perspective, network modeling of genes will be highly useful for understanding functional organizations of key regulators involved in operational pathways of each cell state^12^. Network biology has shifted our perception of a cell from a system mainly comprised of the linear signaling pathways to one occupied by many highly complex intertwined connections among molecules. In particular, the gene regulatory network (GRN) is an intuitive but versatile graph model for functional analysis that has been extensively utilized over the past decade. GRNs have made significant contributions to identifying disease biomarkers and therapeutic targets and were ultimately realized as a crucial tool for deciphering medical genomics data^13^. Scrutinizing the regulatory interactions between genes in various biological contexts will provide valuable insights into how the emergent functions of a given living system was designed to be regulated.

In this review article, we introduce the definition of single-cell network biology and present the current methodologies to infer GRNs from scRNA-seq data and determine how they can improve our understanding of regulatory circuits for cellular identity and facilitate the practice of precision medicine.

## What is single-cell network biology?

Network biology has served as a useful tool for the study of complex cellular systems by providing a glimpse into the functional organization of genes operating in normal and disease states. The GRN is a particularly useful type of gene network that is composed of regulatory relationships inferred from variations across many sources of expression. Typical approaches to analyze GRNs include the identification of hub genes based on network centrality measures^14^ and functional modules using algorithms for finding network communities^15^. Network biology has already proven useful for the study of cellular systems, and here, we present an emerging approach in network biology with single-cell transcriptome data, namely, *single-cell network biology*.

**Fig. 1:**
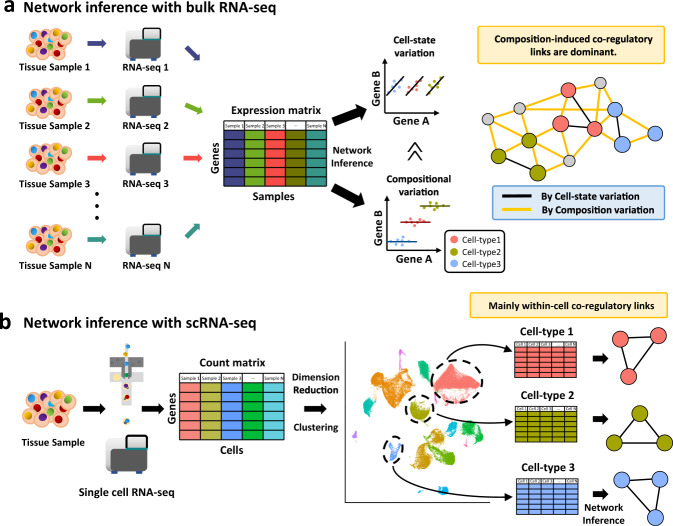
Comparison between network inference with bulk RNA-seq and scRNA-seq. **a** Network inference with bulk RNA-seq analysis. Multiple tissue samples and sequencing are required to produce a gene-by-sample matrix. Correlation between genes can be detected from both expression variation across cell states and variation of cell-type composition across tissue samples. The resultant coregulatory network is mostly composed of cell-type composition-induced coexpression. **b** Network inference with scRNA-seq. A single tissue sample is disassociated into cells that are individually analyzed in parallel. Clustering of the cells along with dimension reduction enables the identification of cell populations for each of the major cell types. Using a gene-by-cell count matrix for each cell type, we can infer networks mainly composed of within-cell coregulatory links.

Before the era of single-cell genomics, transcriptomic data were generated from tissue samples using bulk RNA sequencing (bulk RNA-seq). To estimate expression correlation between genes, a large number of expression measurements was generally required, accordingly demanding an equal number of sequencing reactions for tissue-based analysis. Consequently, the correlation of gene expression could be measured through a *sample-by-gene matrix* (Fig. 1a). Therefore, it is imperative to prepare a large number of samples for network modeling based on bulk RNA-seq data. Conversely, GRNs can be inferred from a single sample preparation followed by a single sequencing reaction with scRNA-seq analysis because it can generate expression measurements for generally hundreds to thousands of individual cells in parallel, generating a *cell-by-gene matrix* (Fig. 1b). To infer regulatory interactions specific to a particular cell type, we need to divide cells into groups representing cell types using dimension reduction and unsupervised clustering. This procedure provides multiple cell-by-gene matrices for distinct cell types, each of which will be used for building cell-type-specific GRNs. Recently, multiple studies demonstrated that the majority of bulk tissue coregulatory links are explained by “*cell-type composition variation*” among samples rather than “*state variation within a cell type*”^16,17^. Therefore, only a fraction of the network inferred from bulk RNA-seq data might represent true within-cell coregulation between genes (Fig. 1a). In contrast, networks inferred from the cell-by-gene matrix for each cell type mainly represent intra-cell-type coregulatory relations between genes (Fig. 1b).

Needless to say, the first benefit of single-cell network biology is its enabling of the reconstruction of cell-type-specific transcriptional regulatory programs. Since the regulatory program specific to each cell type is the core element governing the cellular identity, cell-type-specific GRNs would be key tools for the study of cellular heterogeneity. Furthermore, these cell-type-specific GRNs will reveal key regulatory factors and circuits for specific cell types, facilitating mapping between disease-associated variants and affected cell types. In addition, single-cell network biology provides technical advantages. First, it requires only a small amount of tissue sample for network modeling; even a single biopsy would suffice with adequately high throughput. Second, it can infer regulatory networks from single cells at various levels of cellular identities: major types, subtypes, or states. Third, it can infer regulatory networks from single cells of each person, resulting in personalized GRNs. Thus, in this aspect, single-cell network biology is cost-effective and highly flexible and provides a personalized platform for biomedical research.

### Network inference from single-cell gene expression data

Various algorithms for inferring regulatory interactions between genes using bulk transcriptome data have been developed. Popular approaches to network inference from bulk transcriptome data are based on Boolean networks, Bayesian networks, ordinary differential equations (ODEs), information theory, regression, and correlation^18–20^. Although these methods can be directly applied to single-cell transcriptome data with some adjustment, network inference algorithms specifically developed for single-cell transcriptome data are also available.

Since single-cell transcriptome data can be ordered by pseudotime, many algorithms to infer regulatory networks based on time-ordered transcriptomes have been explicitly developed. The basic assumption of trajectory analysis is that each cell lies in a continuous process of cellular differentiation. The trajectory reconstructed by “pseudotemporal” ordering of cells can then be used for network inference. However, the lack of consensus among resultant trajectories implies that the performance of the network inference with pseudotime information will greatly depend on the trajectory analysis algorithm. Pseudotime information has been used to reconstruct GRNs^21–24^ from single-cell transcriptome data. A recent benchmarking study, however, showed that the methods that do not require pseudotime information performed better^25^.

There are a wide variety of metrics that can be used for measuring coregulatory associations between genes, but their application for single-cell transcriptome data was mostly unsatisfactory^26^. Another benchmarking study concluded that most of the currently available methods for regulatory network inference are not effective for single-cell transcriptome data, even those explicitly developed for single-cell studies^27^. The high proportion of false-positive network links inferred from single-cell gene expression data may be attributable to the intrinsic sparsity and high technical variation. Although these benchmarking results may suggest a lack of general applicability of network inference methods for single-cell biology, caution is advised in making such conclusions. The true positive regulatory links used for evaluation may not accurately represent the ground truth of the regulatory gene network in the tested cell types or states. In addition, the optimal network inference method for given single-cell data could vary across cell types.

As this review focuses on the application of single-cell network biology, we only provide a brief description of major approaches to GRN inference from single-cell transcriptome data. More extensive reviews about computational algorithms are available from other recent publications^28,29^.

#### Boolean models

A Boolean network is the simplest approach to reconstructing regulatory gene networks^30^. In systems biology, a Boolean network refers to a set of genes with binary states (activated or repressed)^31^. This approach is often used to describe the interaction between mRNAs and proteins to predict gene patterns^32^. In this network, each cell is classified into a certain state, and similar cells are then connected. The resulting state-cell graph provides useful information about key regulators that drive certain cellular states. Its simplicity allows the resulting network to be determined with as few assumptions as possible, with one naturally being that all genes must follow a binary law. Single-cell Boolean GRNs have been successfully applied to predict curated models of hematopoiesis^33–35^. A drawback of this approach is the computational burden. Thus, Boolean-based tools have limited scalability, which will prevent users from building a genome-scale network. Therefore, users must carefully select the genes they wish to model, which is usually no more than 100 genes. The Partially Observed Boolean Dynamical system model is a framework for modeling the behavior of GRNs, and this approach allows indirect and incomplete observation of gene states and has been explored for application to scRNA-seq data^36^.

#### Ordinary differential equation (ODE) models

GRN modeling via ODE focuses on a series of discrete states to capture the dynamics of the network in question. While other methods discretize variables, ODE uses continuous variables and is one of the popular methods to map a dynamic system of gene regulation. To date, ODE is the best analyzed approach for nonlinear systems^37^. In this model, the change in expression over continuous time is characterized by a function that takes the inhibitory or activating influence of other genes as variables^18^. This approach is most suitable for identifying a process in a system that is assumed to be continuous (e.g., differentiation). The input time scale could be either an inferred pseudotime or metadata from a time-series experiment. SCODE^38^ is a network construction tool that relies on ODE to map differentiation in single-cell transcriptome data. Some tools based on ODE assume a steady state condition^39,40^, which makes them suboptimal for differentiation-related analysis.

#### Regression models

Most regression-based network inference tools follow an underlying assumption that the expression of all genes can be summarized as a simple weighted linear equation. For this assumption to hold true and produce a reliable prediction, the variables of the data must be independent, and the residuals (errors of fitted linear model) must follow a normal distribution, which is not usually the case for current single-cell transcriptome data. Therefore, most network inference tools based on regression models must be adjusted by a statistical trick (e.g., polynomial modeling, data transformation) to bypass these assumptions. Users must be careful so that this preprocessing step does not compromise the overall structure of the data. In this approach, users may need to provide a list of regulators such as transcription factors (TFs) as input data. Then, the network inference algorithm deconstructs the problem of explaining the expression of a certain target gene with a set of regulators. Here, each subproblem is viewed as a feature selection. Regression-based approaches not only estimate the underlying association between regulators and target genes but also infer the association intensities. The success in ensemble of regression trees (random forest) by GENIE3^41^ has led to this approach being widely used for network inference from both bulk and single-cell transcriptome data. However, GENIE3 calculation is not feasible for data from more than several thousand cells. Subsequently, a much faster and more scalable assembly method for regression trees, GRNBoost, was developed^42,43^. Regulatory networks inferred from single-cell regression analysis tend to have more false-positive links than those inferred by bulk transcriptome regression analysis. To reduce false-positive links, networks inferred from GENIE3 or GRNBoost were filtered for putative direct-binding targets based on TF binding motif enrichment in the SCENIC software package^42^.

#### Correlation and other association models

GRNs based on coregulatory interactions are commonly inferred from correlations between genes across sources of expression variation^44^. Common measures of expression correlation between genes are the Pearson correlation coefficient and rank-based Spearman correlation coefficient. Sources of expression variation are not limited to cell state differences. A large portion of variation can originate from various technical factors, which can easily create confounding effects in correlation inference. Batch effects across samples can also generate nonbiological variation. Because single-cell transcriptomic data are associated with high noise and sparsity, the effect of technical variation could be more critical for single-cell coregulatory network inference. An evaluation of coexpression-based network inference with scRNA-seq data from 31 individual studies comprising 163 cell types showed lower retrieval of known functional links than those inferred from bulk RNA-seq data^45^. The same study also showed reduced performance of coexpression-based network inference with the normalization of UMI data, probably due to unintended covariation, particularly among low-expressing genes. The improved performance with batch-corrected UMI data^45^, however, suggests that with single-cell coexpression-based network inference, extra care is needed for handling technical variations.

Mutual information (MI) can also measure associations between genes based on expression profiles, and it is particularly useful for mapping nonlinear associations^46^. In constructing a coexpression network from scRNA-seq data, users must consider the various technical properties distinct among different sequencing platforms that govern single-cell transcriptome data. An algorithm of MI-based network inference has been explicitly developed for single-cell transcriptome data^47^.

The coregulatory association between genes with multiple sources of expression variations can be measured by many other metrics. Recently, 17 distinct measures of association for inferring gene networks were evaluated and showed that proportionality measures performed best across multiple scRNA-seq datasets and technologies^26^. The compositional nature of transcriptomic data, in which only the relative abundance of transcripts is measured per sample^48^, may contribute to the high performance because scRNA-seq currently only captures a small proportion of the total transcripts per cell. It is, however, noteworthy that all the association measures, including proportionality, assessed in this study barely performed above random expectation, suggesting that the high noise and sparsity of scRNA-seq data must be addressed during data preprocessing before network inference. One such effort recently developed is a method for measuring correlation with scRNA-seq data by pooling cells considered biological replicates and transforming the count matrix to *z* scores, which dramatically increases correlation between genes and facilitates network inference^49^.

### Network filtration for single-cell gene expression

While the “bottom-up approaches” are mainly used to infer cell-type-specific networks from gene expression data, they can also be constructed by filtration of reference gene networks through single-cell gene expression data (referred to as the “top–down approach”). In this approach, single-cell transcriptome data that contain multiple factors are used to fine-tune the reference network to reflect specific context. Gene network databases, such as STRING^50^, HumanNet^51^, and PCNET^52^ provide high-confidence gene functional links. Filtering the global networks for expressed genes for a distinct cell type will result in a cell-type-specific network. The “top–down approach” for constructing context-specific networks with bulk RNA-seq data has already been applied to cancer research. Prognostic biomarkers of ovarian cancer and leukemia have been identified by filtering the global protein–protein interaction network for disease specificity^53^. Sample-specific network^54^ analysis has been shown to be more effective for identifying driver genes in individual tumors^55^, and aggregating these drivers across cancers may reveal new insights into precision cancer therapy.

SCINET^56^ is a recent computational framework that allows optimal filtering of the reference network to obtain a cell-type-specific network according to the input single-cell data. Using these cell-type-specific networks, the authors showed that disease-associated genes tend to interact with each other with cell-type specificity, with marker genes showing higher cell-type-specific centralities than those in the global network by integration of cell-type-specific networks. This analytical framework, which can be generally applied to any reference network and any single-cell expression dataset, enables researchers to infer cell types and cell-type-specific modules governing certain disorders.

### Hypothesis generation in single-cell network biology

Global gene networks inferred from diverse biological contexts have proven useful in generating hypotheses of the functions and phenotypic effects of genes via network centrality and information propagation through the network. Moreover, analysis of network communities can elucidate pathways or functional modules for complex phenotypes such as diseases^57^. Cell-type-specific networks along with single-cell gene expression data can extend the power of network biology to explain the cellular heterogeneity underlying phenotypes of multicellular organisms such as human diseases. Major strategies for hypothesis generation in single-cell network biology (summarized in Table 1) are based on identifying context-associated subnetworks and utilizing topological dynamics. In addition, analysis of personalized gene networks along with genotype information can elucidate network-mediated effects of disease-associated genetic variants.

#### Hypothesis from subnetwork analysis

**Fig. 2:**
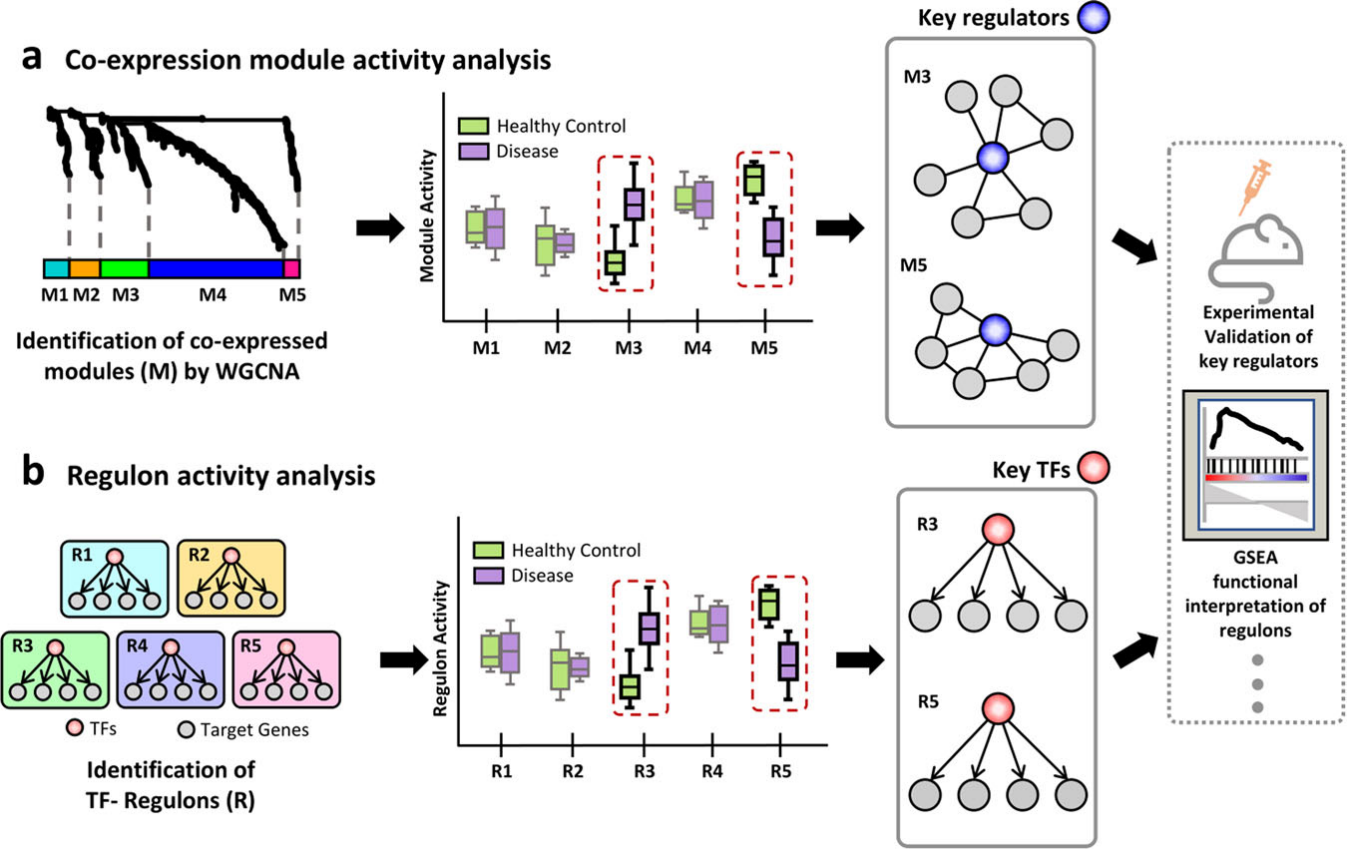
Hypothesis generation from subnetwork analysis in single-cell network biology. **a** Weighted correlation network analysis (WGCNA) of scRNA-seq data generally reveals multiple modules (M1-5) of coexpressed genes of various sizes. The activity of modules can be measured by the average gene expression level. Module activity may significantly differ between cells from different states (e.g., cells of disease samples versus those of healthy controls), which suggests that this coexpressed module is associated with the disease state and may contain key regulators for the disease, often those with high network centrality. **b** Transcription factor (TF)-target interaction inference generates a set of regulons (R1-5) that are genes regulated by each TF. Comparison of regulon activity between healthy and disease states, similar to module activity, can suggest its association with the disease state. Then, the TF for the associated regulon is predicted to be a key regulator. These candidate regulators are often subjected to experimental validation and gene set enrichment analysis (GSEA) for functional interpretation.

Pathways rather than individual genes are the functional units of cells. Thus, pathway-based functional interpretation of cellular states is more intuitive than gene-based interpretation. Weighted correlation network analysis (WGCNA)^58^ has been a popular tool for identifying functional modules based on coexpression networks inferred from a large number of gene expression profiles. WGCNA with single-cell transcriptome data for a cell type may identify functional modules that are associated with a particular state (e.g., disease-related state) of the cell type. Often, by using topological properties (e.g., centrality) or external functional information, we may be able to identify key regulators of functional modules and, in turn, the associated cellular states (Fig. 2a). For example, WGCNA along with scRNA-seq data from early embryo cells revealed that each stage of the early development of mouse and human embryos can be delineated by a few functional modules^59^. WGCNA on single-cell transcriptome data also enabled the discovery of signals that activate dormant neural stem cells in nonneurogenic brain regions^60^, regulators of chemotherapy resistance in esophageal squamous cell carcinoma^61^ and prognostic markers for prostate cancer^62^. The WGCNA package requires users to adjust various parameters so that appropriate modules are defined, and this may often become a potential difficulty in the absence of prior knowledge of disease-associated gene sets.

Subnetworks composed of a TF and its target genes are also useful for functional analysis in single-cell network biology. Here, a set of target genes regulated by each TF is called a regulon. SCENIC^42^ is a popular software tool for the generation of TF-regulon subnetworks for given scRNA-seq data and their downstream analysis. In this analytical platform, individual cells or subpopulations that represent a particular cell state can be depicted by the activity of each regulon. Because each regulon is considered a regulatory unit, regulon activity profiles across cellular states can suggest GRNs governing cellular identity or transitions. Moreover, regulon analysis facilitated the identification of key regulators for cellular states and interpretation of their target pathways by gene set enrichment analysis for the regulon genes (Fig. 2b). In a recent study, regulon-based analysis of scRNA-seq data of patient-derived melanoma cultures revealed key regulators and GRNs specific for intermediate states during the epithelial–mesenchymal transition of melanoma cells^63^, which may provide new therapeutic targets to prevent the acquisition of metastatic potential and drug resistance due to cell state switching.

#### Hypothesis from network topology analysis

**Fig. 3:**
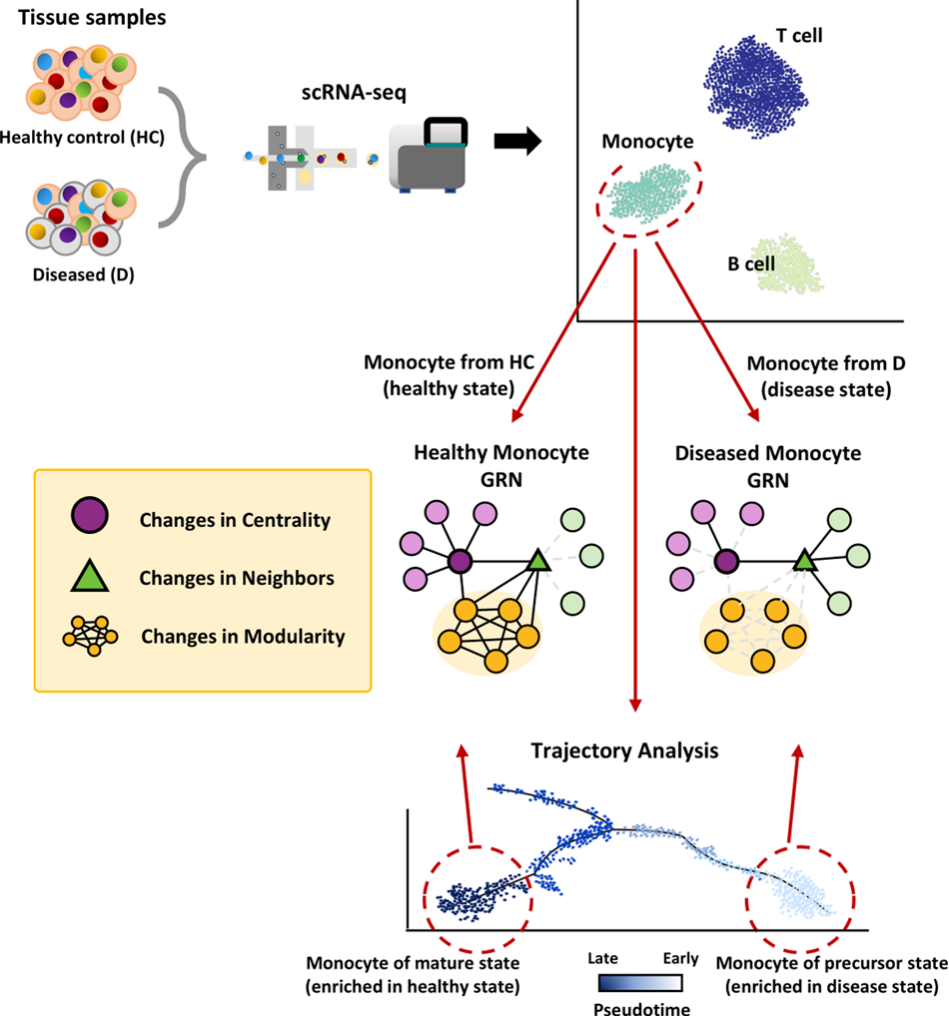
Hypothesis generation from network topology analysis in single-cell network biology. Inferences in coregulatory transcriptome profiles of cells from two distinct states (healthy control versus disease state) lead to the construction of different GRNs. Genes that show changes in three types of network topology are likely to be associated with the state: centrality, neighbors, and modularity. For example, correlation analyses for monocytes from healthy and diseased samples may generate different networks, and changes in three types of topology between them will be examined for every gene. Similarly, networks for different developmental times along with topological analysis would suggest disease-associated genes because many disease states are associated with defects in development. For example, defects in the maturation of monocytes into functional dendritic cells would result in immune disorders.

Emergent cellular phenotypes depend not only on genotypes but also on edgotypes, context-specific networks of molecular interactions^64^, implying that the dynamics of regulatory interactions underlie cellular heterogeneity. Comparisons between cell-type-specific networks for different states, such as disease and healthy states, will show topological changes for each gene in centrality (hubness) and neighbors (targets). Genes that show significant changes in one of these topological properties would be candidate regulators involved in the cellular state of interest (Fig. 3). For example, a recent study generated healthy and type 2 diabetes (T2D) regulatory networks using scRNA-seq data from pancreatic islet cells^49^. The study demonstrated that many genes with significant changes in centrality are involved in T2D. Another study generated GRNs for self-renewing cells, erythroid-committed progenitors, and myeloid-committed progenitors and demonstrated that the lineage regulator DDIT3 changes its targets in three different GRNs^65^. Gene sets involved in particular biological processes or diseases may also change their modularity (intraconnectivity) between different cellular states, which suggests their association with a particular cellular state (e.g., disease-related state). For example, gene networks were generated for six brain cell types^56^, in which neuropsychiatric disorder genes were found to preferentially interact in neuronal cells, whereas genes for neurodegenerative diseases do so in glial cells. Another recent study demonstrated that modularity measures based on the enrichment of coexpression among genes associated with specific neurodevelopmental disorders increased in specific cell types^66^. These results suggest that disease-related genes tend to preferentially interact with cell types for different disease classes. Although network topology analysis offers an intuitive method for observing a cell-type-specific system, a large number of links and the associated complexity potentially cause difficulty in interpretation. Researchers must also take into account that many experimental and technical factors must be controlled to accurately compare different networks.

#### Hypothesis from genotype-network association

**Fig. 4:**
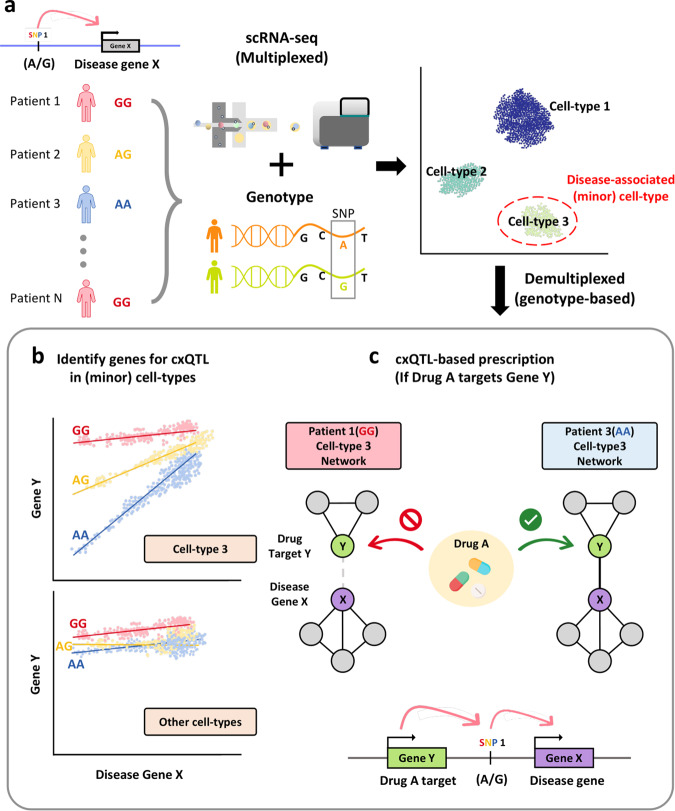
Hypothesis generation from genotype-network association in single-cell network biology. **a** Many disease-associated single nucleotide polymorphisms (SNPs), which are called expression QTLs (eQTLs), exert phenotypic effects through the regulation of gene expression in a cell type-specific manner. Therefore, eQTL analysis needs to be conducted for specific cell types, particularly for minor cell types. The recently developed multiplexed scRNA-seq technology along with demultiplexing based on genotype information will facilitate cell-type-specific eQTL mapping. **b** Some eQTL effects are dependent on the expression of other genes. This dependency is detected by genotype-specific coexpression, called coexpression QTL (cxQTL). Here, a disease gene X is coexpressed with gene Y only if its eQTL has a homozygous major allele (AA). **c** If the gene Y is a target of drug A that eventually inhibits the activity of disease gene X via interaction with gene Y, the genotype-dependent coregulatory interaction between genes X and Y is critical for drug action. Then, for prescription of drug A, the cxQTL genotype information can be utilized for precision medicine (e.g., prescribing it only for patients with SNP AA).

A major problem in health care today is imprecision medicine, wherein only a small portion of patients respond to routinely prescribed drugs^67^. This may be because patients have different genetic variations that influence the functional effects of genes involved in pathogenesis or pharmacodynamics. The majority of such variations exert phenotypic effects via the action of expression quantitative trait loci (eQTLs)^68^ because most of them are located within noncoding regions^69^. The eQTLs have long been suggested to exert their influence in a cell-specific manner, and the large portion of unresolved eQTLs may be attributable to the cell-type dependent effects of these eQTLs^70,71^. Cell-type-specific eQTL analysis can be conducted by sorting each cell type, which generally has a high cost. As scRNA-seq can provide transcriptome data for multiple cell types of a given tissue simultaneously, it can greatly facilitate cell-type-specific eQTL analysis^72–75^ (Fig. 4a). Cell-type-specific eQTL studies may possibly reduce the detection of false-positive SNPs associated with disease that have often emerged as potential limitations of bulk RNA-seq-based eQTL research (e.g., Simpson’s paradox). Moreover, utilizing a large number of cells in single-cell datasets may significantly reduce the number of samples required for eQTL detection. It is noteworthy, however, that for more accurate analysis, this approach will require a larger number of donors than typical single-cell-based studies.

**Table 1:** Summary of hypothesis generation through single-cell network biology.

Approaches	Advantages	Limitations	References
Subnetwork analysis of module and regulon activities (Fig. 2)	Enabling the identification of key regulators that are associated with a disease-associated phenotype at cell-type resolutions	1. Various parameters to adjust for module identification. Difficult to choose optimal parameters without some form of prior knowledge of functional gene sets. 2. No definitive method exists for detecting regulatory links. Inferred links will vary depending on applied network-inference algorithm.	^42,58^
Topology analysis of disease-associated cell-type-specific network (Fig. 3)	Graph-based, intuitive, and comprehensive methods for prioritization of genes associated with a disease-associated phenotype at cell-type resolutions	1. Various measures of network centrality (hubness). Different centrality measures may predict different candidate genes. 2. Various experimental and technical factors must be taken into consideration that might affect network topological changes.	^49,56^
Genotype-network association and coexpression QTL analysis (Fig. 4)	1. Considerable amount of false-positive SNPs may be removed from cell-type specificity. 2. Significantly fewer number of samples needed compared to eQTL studies through the bulk counterpart.	1. Compared to other types of single-cell studies, relatively large number of patient samples may be necessary. 2. Doublets (two genotypes barcoded in a single cell) must be taken into consideration and processed during demultiplexing of data.	^77^

Interestingly, some eQTL effects of a gene can be modified by the expression of another gene^76^ (Fig. 4b). For example, the effect of a *FADS2* eQTL is modulated by the expression of the sterol binding factor gene *SREBF2*. Therefore, these genetic variants are called *coexpression QTLs*, because they affect the coregulatory relationship between two genes^76,77^. Single-cell transcriptome data from each person can be sufficient to infer gene–gene correlation, building personalized GRNs^77,78^. Given that personal- and cell-type-specific coregulatory relationships between genes can be modeled using scRNA-seq data, we may test whether personal genetic variants affect disease risk or drug response by altering coregulatory interactions. If a coregulatory interaction between a disease gene and a drug target that affects the disease gene activity is modulated by a coexpression QTL, this genotype information could be utilized in tailored prescription for individual patients in the future (Fig. 4c).

### Challenges and future perspectives

The major challenges in single-cell network biology are associated with the single-cell omics technology, as the quality of inferred networks relies largely on the quality of single-cell transcriptome data. Single-cell profiling technologies are rapidly evolving. However, various technical hurdles, such as low capture efficiency, high dropout rates, and high noise in signals, must be considered and overcome to observe true biological variations in gene expression^6^. New computational methods need to be developed to overcome those intrinsic limitations of scRNA-seq data. For example, imputation of dropouts in single-cell transcriptome data will vary in the probability of false gene–gene correlations^79^, and the methods need to be further improved in the future. In addition, the integration of multimodal single-cell omics data^80^ and multiomics data^81^ would contribute in improving network inference and interpretations.

Many statistical approaches have been developed to address these issues, and depending on the basic assumptions that the researchers are willing to adhere to an appropriate method must be chosen for different datasets. Each algorithm with its own preprocessing steps will result in different networks. Therefore, preprocessing of the single-cell dataset will be the critical step of the network inference algorithm. Moreover, network inference tools with different algorithmic concepts will perform optimally for different sets of data (e.g., time series, developmental, perturbation). Therefore, researchers must choose their methods depending on the data that they have collected and the system that they wish to evaluate. Different types of networks (regulatory or functional) will provide different insights, and it is important to extract reasonable conclusions allowed from numerous types of networks and make suitable predictions.

In this paper, we highlighted the effectiveness of using network-based studies in resolving cellular heterogeneity. Personalized gene networks obtained from single-cell transcriptome data will facilitate the development of novel applications based on personal genetic variation for precision medicine. For translation of single-cell network analysis to clinical settings, user-friendly analytical pipelines must be established for different types of diseases. These efforts together will improve our ability to accurately diagnose and predict disease risks and ultimately lead to the development of precision medicine.
